# Annexin A9 promotes cell proliferation by regulating the Wnt signaling pathway in colorectal cancer

**DOI:** 10.1007/s13577-023-00939-x

**Published:** 2023-06-22

**Authors:** Xuemei Lu, Liqiang Hu, Jiayan Mao, Shufen Zhang, Ying Cai, Wei Chen

**Affiliations:** 1 College of Pharmaceutical Sciences, Zhejiang Chinese Medical University, Hangzhou, 310053 Zhejiang China; 2 Cancer Institute of Integrated Traditional Chinese and Western Medicine, Key Laboratory of Cancer Prevention and Therapy Combining Traditional Chinese and Western Medicine of Zhejiang Province, Zhejiang Academy of Traditional Chinese Medicine, No. 234, Gucui Road, Hangzhou, 310012 Zhejiang China

**Keywords:** ANXA9, Colorectal cancer, Prognosis, Immune infiltration, Wnt signaling pathway

## Abstract

Colorectal cancer (CRC) is one of the leading causes of cancer-related mortality worldwide. Expression of Annexin A9 (ANXA9), a member of the annexin A family, is upregulated in CRC. However, the molecular role of ANXA9 in CRC remains unknown. In the present study, we aimed to investigate the function of ANXA9 and to elucidate the mechanisms underlying its regulation in CRC. In this study, mRNA expression data and clinical information were downloaded from The Cancer Genome Atlas (TCGA) and GEPIA database, respectively. Kaplan–Meier analysis was used to analyze the survival rates. LinkedOmics and Metascape databases were used to explore the potential mechanisms of regulation of ANXA9 and to identify genes co-expressed with ANXA9. Finally, in vitro experiments were used to evaluate the function of ANXA9 and explore potential mechanisms. We found that ANXA9 expression was significantly elevated in CRC tissue and cells. High ANXA9 expression was associated with shorter overall survival, poorer disease specific survival, as well as with patient age, clinical stage, M stage, and OS events in CRC. Knockdown of ANXA9 inhibited cell proliferation, invasion, migratory potential, and cell cycle arrest. Mechanistically, functional analysis revealed that genes co-expressed with ANXA9 were mainly enriched in the Wnt signaling pathway. ANXA9 deletion suppressed cell proliferation via the Wnt signaling pathway, while Wnt activation reversed the effects of ANXA9. In conclusion, ANXA9 may promote CRC progression by regulating the Wnt signaling pathway and may be a potential diagnostic biomarker in the clinical management of CRC.

**Supplementary Information:**

The online version contains supplementary material available at 10.1007/s13577-023-00939-x.

## Introduction

Colorectal cancer (CRC) is a major cause of cancer death [1]. Despite improvement in early diagnosis, prevention and therapeutic options, CRC remains a disease with high incidence and mortality [2, 3]. In 2020, the survey of International Agency for Research on Cancer (IARC) (https://www.iarc.who.int/) concluded that CRC accounted for approximately 10% of the total cancer cases, after breast cancer (11.7%) and lung cancer (11.4%), and determined that CRC is the a leading cause of cancer mortality worldwide (9.4%), second only to lung cancer (18%). Therefore, to improve patient outcomes, it is essential and urgent to find new prognostic biomarkers and effective therapeutic targets.

Annexins are a group of calcium-dependent phospholipid-binding proteins. At least 1000 Annexin subfamily members have been identified [4]. Annexins are divided into five categories: annexin A (ANXA), annexin B, annexin C, annexin D, and annexin E [5]. The ANXA family mainly exists in vertebrates [5]. The ANXA family consists of 12 members, including ANXA1-ANXA11 and ANXA13 [6]. Ectopic expression of ANXA family members is associated with tumorigenesis and progression in many cancers. For example, ANXA1 can promote the growth of breast tumors, reduce patient survival, and is associated with the suppressive function of Treg cells [7]. ANXA2 potentiates migration, invasion ≤ and metastasis in esophageal cancer by activating MYC-HIF1A-VEGF signaling [8]. ANXA2 acts as a target of miR-185 and exhibits oncogenic functions in glioblastoma multiforme [9]. Low ANXA10 expression is associated with unfavorable prognosis and acts a clinically relevant marker for predicting outcome in bladder cancer [10].

ANXA9 is a member of ANXA family, and patients with CRC that exhibits high expression of ANXA9 have poor prognosis, suggesting that ANXA9 expression is a prognostic factor in CRC outcomes [11]. Yu et al*.* demonstrated that ANXA9 may promote the invasion and metastasis of CRC by regulating invasion- and metastasis‑associated genes [12]. However, the molecular regulatory mechanism of ANXA9 in CRC requires additional clarification.

In this study, we examined ANXA family member expression levels and determined the prognostic value associated with ANXA expression in CRC, using multiple databases. We found that ANXA9 is upregulated in CRC, and that high ANXA9 expression is significantly associated with poor overall survival. Subsequently, we determined the association of ANXA9 expression with clinical characteristics and immune infiltration in CRC. Finally, we found that knockdown of ANXA9 significantly inhibited the proliferation of CRC cells in vitro via regulating the Wnt signaling pathway. These results suggest that ANXA9 is significantly upregulated in CRC, and may represent a promising therapeutic biomarker for CRC.

## Materials and methods

### Data acquisition

The TCGA database (https://portal.gdc.cancer.gov) [13] was used to download high-throughput sequencing data from the TCGA-colon adenocarcinoma (TCGA-COAD) and TCGA-rectal adenocarcinoma (TCGA-READ) cohorts. The TCGA-COADREAD cohort comprised of 647 CRC tissue samples and 51 adjacent normal tissue samples. Gene Expression Profiling Interactive Analysis (GEPIA) (http://gepia.cancer-pku.cn/index.html) [14] is a public database that includes 9,736 tumor and 8587 normal samples from the TCGA and the GTEx projects. GSE9348 and GSE156355 were obtained from the Gene Expression Omnibus (GEO) database [15]. The GSE9348 dataset, based on GPL570, included 6 CRC samples and 6 normal samples while the GSE156355 dataset, based on GPL21185, contained 70 CRC samples and 10 normal samples. In this study, we used data from these multiple databases to analyze the gene expression levels between tumor and control samples. We also obtained corresponding clinical information from the TCGA-COADREAD cohort. Because these databases are open to public and freely available, additional approval from the ethics committee is not required.

### Survival analysis

Survival data from clinical samples was obtained from the TCGA-COADREAD cohort. Survival analysis was performed by using the R packages “survival” and “survminer” to plot Kaplan–Meier survival curves, which represented the overall survival (OS) and disease specific survival (DSS) between high and low ANXA2/3/4/9 groups. The Kaplan–Meier method and log-rank test were used for survival analyses.

### Analysis of ANXA9 gene co-expression and functional enrichment

LinkedOmics (http://www.linkedomics.org/login.php) [16] is a publicly available database for analyzing multi-omics data, including 32 TCGA cancer types and 10 Clinical Proteomics Tumor Analysis Consortium (CPTAC) cancer cohorts. We used LinkedOmics to identify the genes co-expressed with ANXA9 in CRC. We selected 123 genes related to ANXA9 expression and subsequently used Metascape (https://metascape.org/) to analyze the pathway enrichment of these genes by gene ontology (GO) and Kyoto Encyclopedia of Genes and Genomes (KEGG) [17]. Only terms with *p* < 0.01, minimum count > 3, and enrichment factor > 1.5 were considered significant.

### Cell culture and transfection

Four human CRC cell lines, including LoVo, SW480, HCT116, and HT29, and a normal human colon epithelial cell line, NCM460, were purchased from the Procell Life Science & Technology Co., Ltd. All cells were cultured in RPMI 1640 medium or DMEM (Gibco; Thermo Fisher Scientific, Waltham, MA, USA) with 10% fetal bovine serum (Gibco; ThermoFisher) at 37 °C in a 5% CO_2_ atmosphere.

HT29 and HCT116 cells were transfected with siRNA targeting ANXA9 (si-ANXA9-1, si-ANXA9-2, si-ANXA9-3) or negative control siRNA (Gene Pharma, Suzhou, China) using OPTI-MEM (Invitrogen; Thermofisher) and Lipofectamine 2000 (Invitrogen; ThermoFisher) according to the manufacturer’s instructions. The ANXA9 siRNA sequences used were as follows: si-ANXA9-1:5′-GGCAGCUCAUCUCACGAAATT-3′; si-ANXA9-2: 5′- GGACGUGGCCAUUGAAAUUTT-3′; si-ANXA9-3: 5′- GCAGUCUACAAACACAAUUTT-3′.

### RNA extraction and quantitative real-time PCR (qRT-PCR)

Total RNA extraction and qRT-PCR were performed as previously described. The 2^−ΔΔCt^ method was used for calculating differences in gene expression levels, and GAPDH served as reference. The following primers were used: ANXA9: forward: 5′-TGAGCCCAATTACCAAGTCC-3′, reverse: 5′-GTTCAGCCAAACACGGAAAT-3′; Homo-ACTB, forward: 5′-TGGCACCCAGCACAATGAA-3′, reverse: 5′- CTAAGTCATAGTCCGCCTAGAAGCA-3′.

### Colony formation and Edu assay

CRC cell proliferation was measured by colony formation and Edu assays. For colony formation assay, si-ANXA9-transfected HT29 and HCT116 cells were plated onto 6-well plates at a density of 1000 cells per well and incubated. After two weeks, the cells were fixed with 4% paraformaldehyde for 15 min and stained with a crystal violet solution for 20 min. Then the number of colonies was counted. For the Edu assay, the transfected cells were seeded on a 24-well plate and cultured for 48 h. After being incubated with 10 µM EdU (Invitrogen) for 2 h at 37 °C, the cells were fixed, permeabilized, and stained with EdU and DAPI. Finally, the EdU-positive cells were observed using fluorescence microscopy.

### Wound healing assay and Transwell invasion assay

Transfected HCT116 and HT29 cells were seeded at a density of 20,000 cells per well in six-well plates. A wound was scratched in the cell monolayer with a sterile pipette tip after the cells reached 80% confluence. Cells were washed with PBS, and cultured in serum-free medium. The width of the wound was photographed and recorded at 0 and 48 h using a light microscope.

Cell invasion ability was determined by Transwell invasion assay. Transfected HCT116 and HT29 cells (5000 cells) were cultured in the upper chamber of 24 well Transwell plate in serum-free medium, and 700 μL of medium with 10% serum was added to the lower chamber. After 48 h, the cells on the bottom surface of the upper Transwell chamber were fixed, stained and photographed. Stained cells were counted in five randomly selected fields under a light microscope.

### Cell cycle assay

Transfected HCT116 and HT29 cells were harvested 48 h after transfection and fixed with 70% cold ethanol overnight. Then cells were incubated in the dark with 400 µl PI staining solution for 30 min. Finally, cell cycle was evaluated by cell flow cytometer (BD Biosciences, San Jose, CA, USA).

### Western blotting

Western blot and protein band detection were performed as reported in a previous study [18]. The antibodies used were as follows: anti-E-cadherin (3195 s, CST), ANXA9 (15,416-1-AP, Wuhansanying, China), anti-Vimentin (3390S, CST), anti-cMYC (13116 s, CST), anti-β-catenin (SAB5700050, Sigma-Aldrich), Snail (26,183-1-AP, Wuhansanying), anti-GAPDH (14C10, CST).

### Statistical analysis

Each in vitro experiment was performed in triplicate. Results are expressed as mean ± SD. GraphPad Prism 7.0 was used for statistical analysis. Survival analysis was performed using the Kaplan–Meier analysis with the log-rank test. Student’s *t*-test or one-way ANOVA was used to identify significant differences between two groups or among multiple groups. A *p*-value of *P* < 0.05 was considered statistically significant.

## Results

### Differential expression of ANXA family members in patients with CRC

**Fig. 1:**
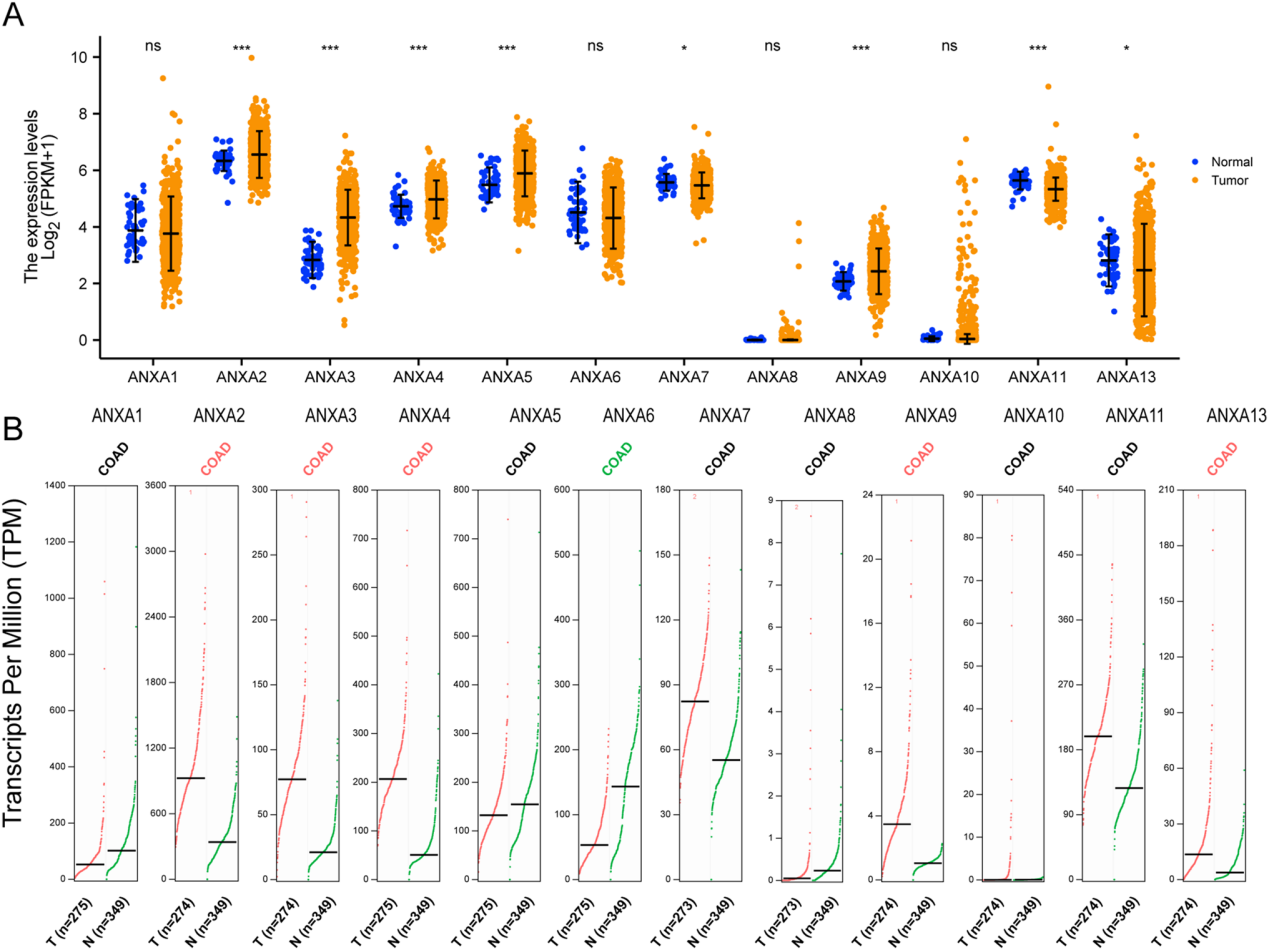
mRNA expression levels of ANXA family genes in CRC. **A** mRNA expression levels of the 12 ANXA family members in CRC tissues and normal tissues from the TCGA. *P < 0.05, ***P < 0.001. **B** mRNA expression levels of 12 ANXA genes in CRC tissue and normal tissue from the GEPIA2 databawse. T: CRC tissues; N: normal tissues

**Fig. 2:**
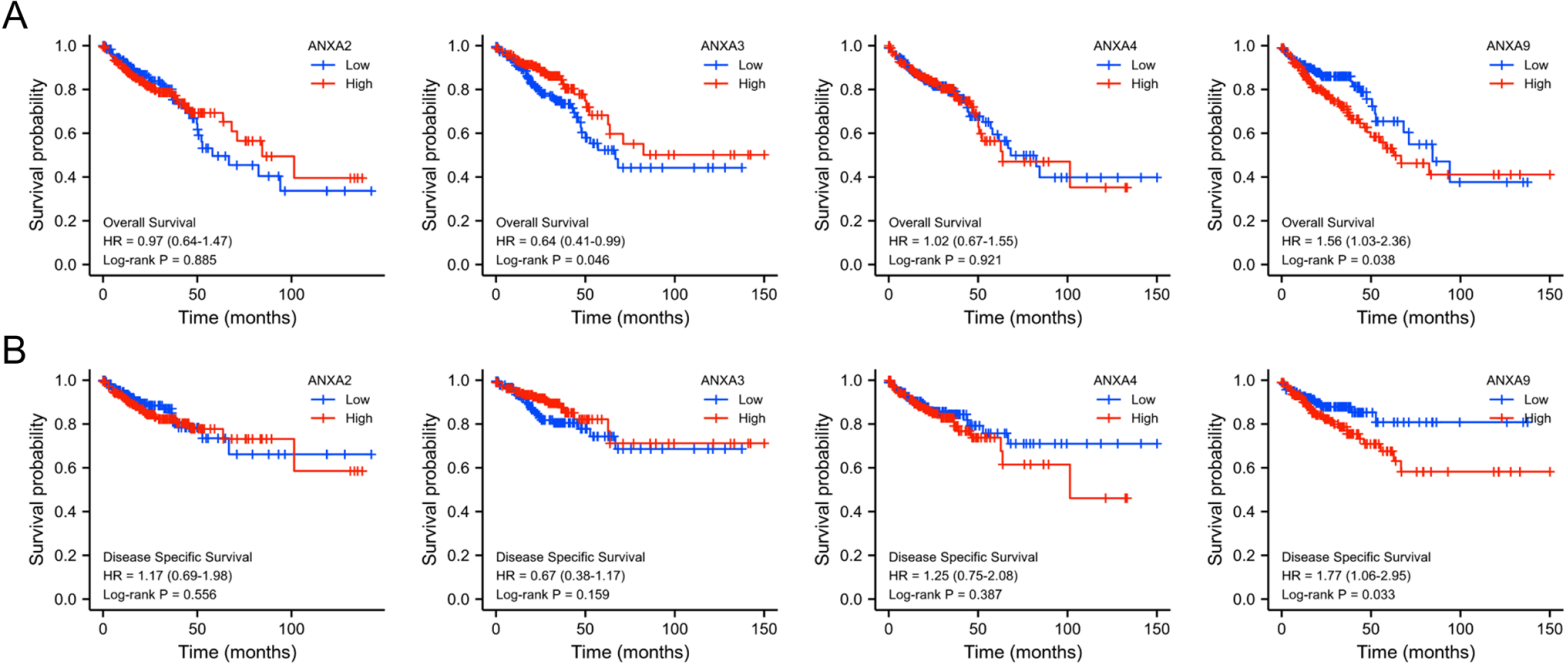
Prognostic value of the mRNA expression of ANXA genes in patients with CRC. **A** OS and **B** DSS curves comparing all CRC patients stratified by high and low expression of ANXA2/3/4/9 (K-M Plotter)

We first investigated the expression of ANXA family members in patients with CRC in the TCGA database. We found that expression of ANXA2, ANXA3, ANXA4, ANXA5, and ANXA9 was elevated in CRC tissues, while expression of ANXA11 and ANXA13 was lower in CRC tissues. However, ANXA1, ANXA6, ANXA7, ANXA8, and ANXA10 expression showed no statistical difference between CRC tissue and normal tissue (Fig. 1A). Further exploration of the expression of ANXA family members in CRC was performed using the GEPIA2 database, which revealed that expression levels of ANXA2/3/4/9/13 were elevated in CRC, and ANXA6 levels were reduced in CRC tissue compared with normal tissue. Analysis of the GEPIA2 databases revealed no significant difference in expression of ANXA5/7/8/10/11 between CRC tissue and normal tissue (Fig. 1B). In addition, we also found an increase of ANXA1/2/3/4/5/9/10 expression in CRC tissues using the ULCAN database (Figure S1). Next, we focused on ANXA2/3/4/9, which were found to echibit higher expression in CRC in the three databases. We further analyzed the correlation between ANXA2/3/4/9, OS and DSS through Kaplan–Meier survival analysis and log rank test analysis. ANXA3 and ANXA9 were significantly correlated with OS (Fig. 2A), and ANXA9 was significantly correlated with DSS (*P* < 0.05) (Fig. 2B). Collectively, these data showed that ANXA9 may be significant in CRC. Therefore, we selected to evaluate ANXA9 in subsequent experiments.

### ANXA9 expression is increased in CRC

**Fig. 3:**
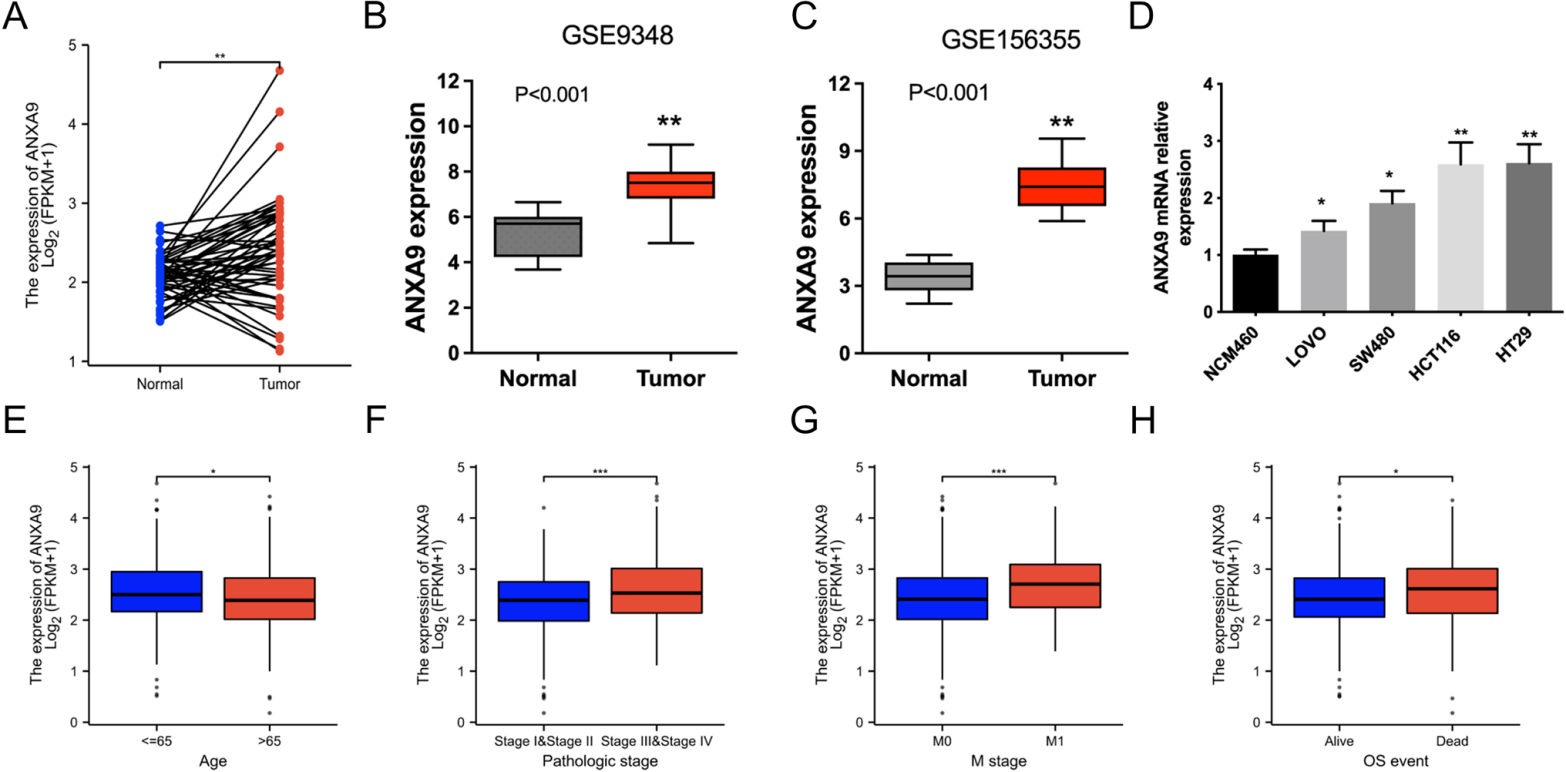
Increased ANXA9 expression in CRC tumors and cells and the association of ANXA9 gene expression with clinical characteristics in CRC patients. **A** ANXA9 expression in CRC samples and paired adjacent non-cancer samples. **B**–**C** ANXA9 expression in samples from the GSE9348 and GSE156355 datasets. **D** ANXA9 expression in CRC cells. Associations between ANXA9 mRNA expression and clinical characteristics in CRC patients based on age (**E**), pathologic stage (**F**), M stage (**G**), OS event status (**H**). *P < 0.05, **P < 0.01, ***P < 0.001

We compared ANXA9 mRNA expression in CRC and paired normal tissue samples using the TCGA-COADREAD cohort. ANXA9 expression was significantly higher in 50 CRC samples than in matched normal samples (Fig. 3A). We further validated these results using the GEO database (GSE9348 and GSE156355). In the GEO database, *ANXA9* expression was elevated in CRC tissue compared with normal tissue (P < 0.001) (Fig. 3B–C). Additionally, ANXA9 expression was higher in CRC cells than that in NCM460 cells (Fig. 3D). Next, to investigate the relation between ANXA9 expression and clinical parameters, we separated the samples of TCGA-COADREAD cohort into a high expression group and a low expression group, according to the mean value of ANXA9 expression (Table S1). ANXA9 mRNA expression was significantly correlated with age, pathologic stage, M stage, and OS event status (Fig. 3E–H; *P* < 0.05). These results demonstrate that ANXA9 expression is significantly upregulated in CRC.

### The relationship between ANXA9 expression and immune infiltration levels in CRC

ssGSEA was used to calculate the correlation between immune cell infiltration and ANXA9 expression in CRC. There were significant differences in 24 immune response gene sets between the high ANXA9 expression group and the low ANXA9 expression group. These gene sets include Th2 cells, cytotoxic cells, activated dendritic cells (aDC), Th1 cells, neutrophils, Treg, NK CD56dim cells, B cells, dendritic cells (DC), Macrophages, T helper cells, Tgd (T gamma delta), Tem (T effector memory), TFH (T follicular helper), NK CD56bright cells, iDC (immature DC), pDC (plasmacytoid DC), mast cells, CD8 T cells, Th17 cells, Tcm (T central memory), eosinophils, and NK cells. ANXA9 expression was positively related to the abundance of Tcm, and negatively associated with the abundance of Th2 cells, cytotoxic cells, aDC, T cells, Th1 cells, Treg, B cells, DC cells, NK CD56bright cells, TFH, T helper cells, CD8 T cells, neutrophils, iDC, macrophages, and mast cells (*P* < 0.05; Fig. S2-A). We also used the TIMER database to further investigate the correlation between ANXA9 expression and immune infiltration. ANXA9 expression was significantly negatively correlated with the level of infiltrating B cells (Fig. S2-B; Cor =   −  0.122, *P* = 1.41e  −  02), CD8^+^ T cells (Cor =  − 0.152, P = 2.07e − 03) and dendritic cells (Cor =   −  0.136, P = 6.4e − 03), but positively correlated with the levels of macrophages (Cor = 0.127, P = 1.03e − 02) in CRC. ANXA9 was negatively correlated with ImmuneScore and EstimateScore but not with StromalScore (Fig. S2-C). In addition, to investigate the relationship between ANXA9 and immune infiltration, we performed the immunohistochemical staining. The results showed that ANXA9 expression was higher in tumor tissues than that in normal tissues and ANXA9 expression level inversely related to the expression level of CD3 and CD68 (Fig. S2d).

### ANXA9 knockdown inhibits the growth of CRC cells in vitro

**Fig. 4:**
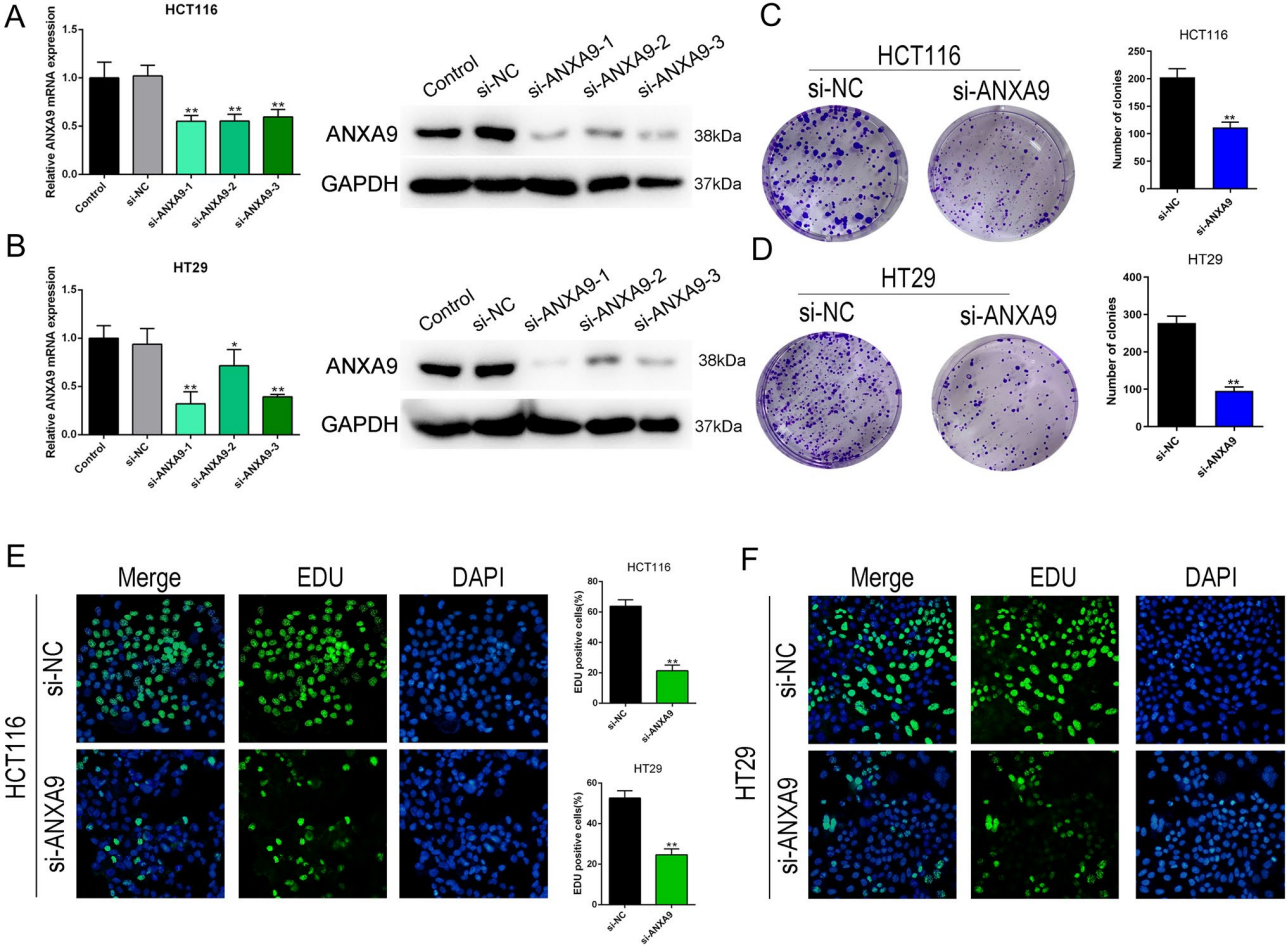
ANXA9 knockdown inhibits the growth of CRC cells in vitro. **A–B** qRT-PCR and western blot was performed to identify the siRNA knockdown efficiency. *P < 0.05; **P < 0.01. **C–D** The effects of ANXA9 knockdown on the colony formation ability of CRC cells was assessed by crystal violet staining. **P < 0.01. **E**–**F** The effect of ANXA9 knockdown on the proliferation of CRC cells was evaluated by EDU assay. **P < 0.01

**Fig. 5:**
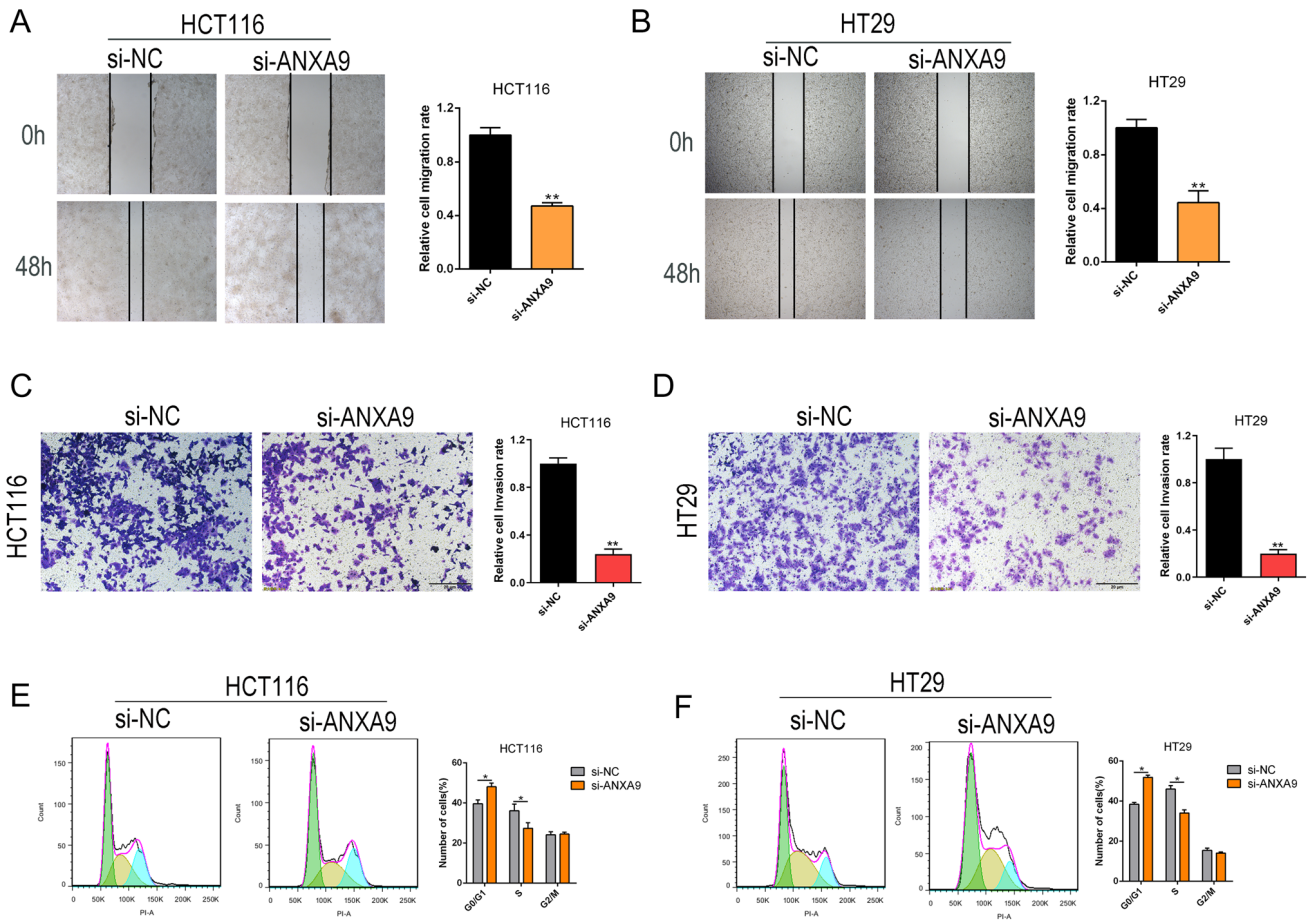
*ANXA9* knockdown suppresses the cell migration, invasion, and cell cycle of CRC cells. **A–B** Wound-healing assays were performed to evaluate CRC cell migration after ANXA9 knockdown (magnification: 200 ×). **P < 0.01. **C–D** Transwell assays were performed to assess CRC cell invasion after ANXA9 knockdown (magnification: 200 ×). **P < 0.01. **E–F** Flow cytometry was performed to examine changes in the cell cycle after ANXA9 knockdown. *P < 0.05

To explore the function of *ANXA9 *in vitro, ANXA9 was knocked down by siRNA (si-ANXA9-1, si-ANXA9-2, si-ANXA9-3) in HCT116 and HT29 cells. qRT-PCR and WB were performed to identify the knockdown efficiency (Fig. 4A–B), and si-ANXA9-1 exerted the most significant knockdown. Therefore, we chose si-ANXA9-1 to knockdown ANXA9 in subsequent experiments. ANXA9 knockdown in HCT116 and HT29 cells resulted in significantly decreased cell growth, as determined by crystal violet assay (Fig. 4C–D). A similar finding was confirmed by EDU assay (Fig. 4E–F). Moreover, wound healing and transwell assays showed that ANXA9 knockdown decreased the invasion and migratory capability of CRC cells (Fig. 5A–D). In addition, the ANXA9 knockdown increased the percentage of CRC cells in G1 and decreased the percentage of cells in the S phase, as determined by flow cytometry (Fig. 5E–F). These data indicate that ANXA9 knockdown inhibits the growth of CRC cells.

### Functional analysis of ANXA9 in colorectal cancer

**Fig. 6:**
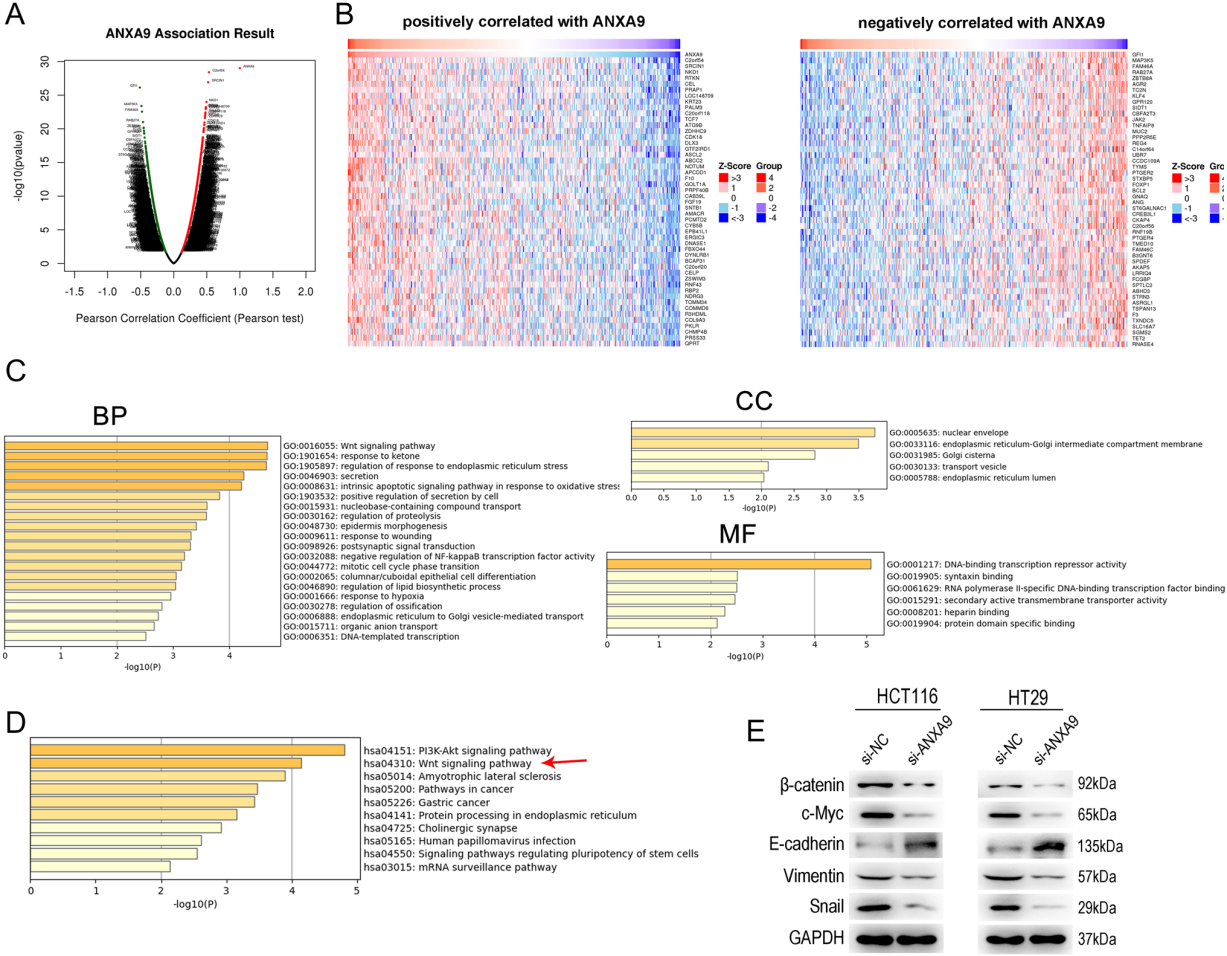
Functional enrichment analysis of genes co-expressed with ANXA9 in CRC. **A** Genes co-expressed with ANXA9 in CRC were determined by Spearman correlation. Red and green dots represent positively and negatively correlated genes, respectively. **B** ANXA9 was positively or negatively correlated with the first 50 genes in the heat map. **C** Results of the GO enrichment analysis. **D** Results of the KEGG pathway enrichment analysis. **E** The protein levels in CRC control and ANXA9 knockdown cells

To investigate the mechanisms underlying the role of ANXA9 in CRC, we used LinkedOmics to analyze the genes that are co-expressed with ANXA9 in CRC. A total of 8447 genes were significantluy correlated with ANXA9: 4158 genes were positively correlated with ANXA9 and 4289 genes were negatively correlated with ANXA9 (FDR < 0.05; Fig. 6A). We visualized the top 50 genes with a significant positive or negative association with ANXA9 (Fig. 6B). We screened the genes based on |R|> 0.4 and FDR < 0.05, and we identified 123 genes co-expressed with ANXA9: 90 positively related genes and 33 negatively related genes. To investigate potential mechanisms involved, we performed GO and KEGG analyses for the 123 co-expressed genes (Fig. 6C–D, respectively). GO molecular functions were mainly enriched in DNA-binding transcription repressor activity, syntaxin binding, and RNA polymerase II-specific DNA-binding transcription factor binding. GO cellular components were mainly enriched in the nuclear envelope, endoplasmic reticulum-Golgi intermediate compartment membrane, transport vesicle, and endoplasmic reticulum lumen. GO biological processes were mainly enriched in the Wnt signaling pathway, secretion and regulation of proteolysis (Fig. 6C). KEGG enrichment analysis identified that the genes co-expressed with ANXA9 in CRC were enriched in the PI3K-AKT pathway, the Wnt signaling pathway, the amyotrophic lateral sclerosis, signaling pathways regulating pluripotency of stem cells, and the mRNA surveillance pathway (Fig. 6D).

### ANXA9 promotes cell proliferation by regulating the Wnt signaling pathway

**Fig. 7:**
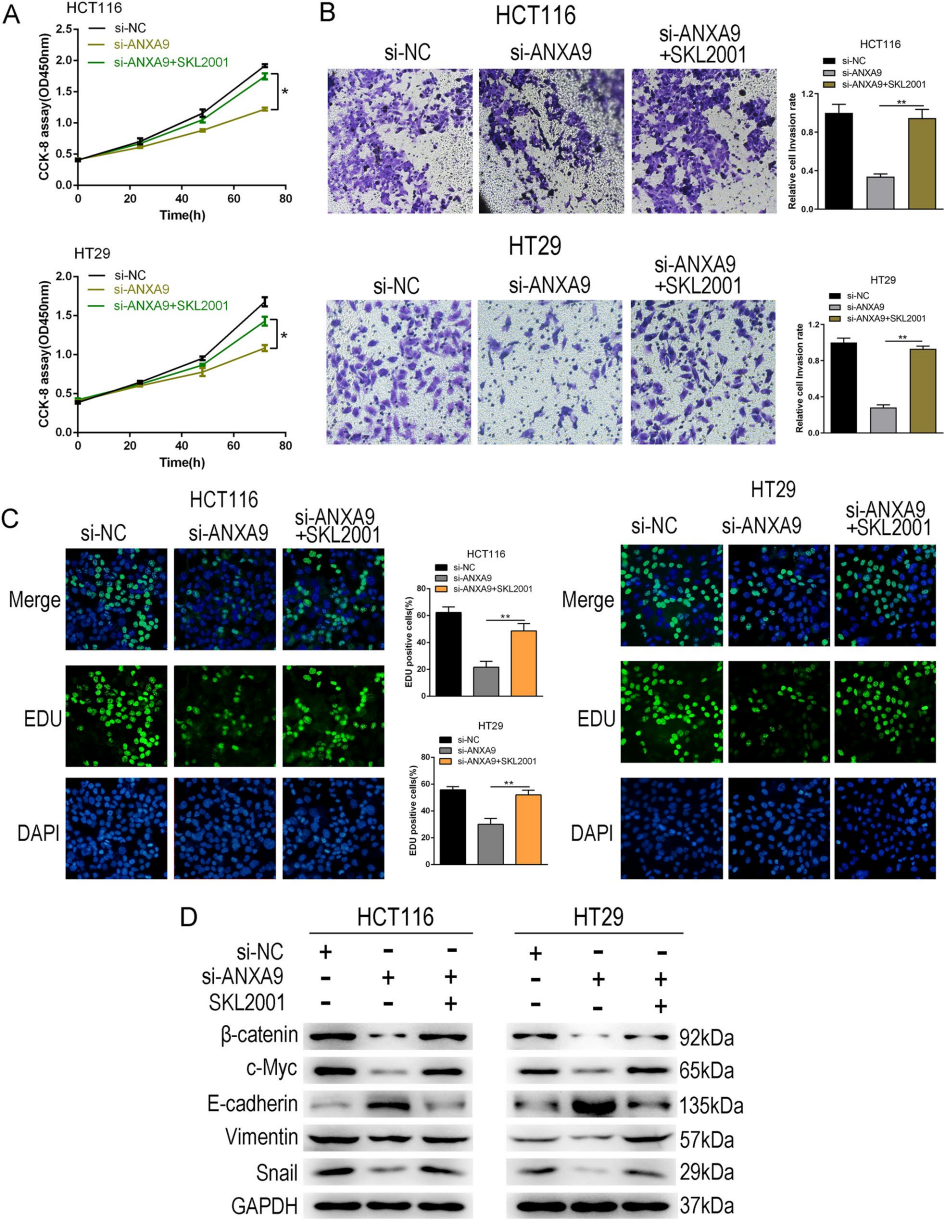
ANXA9 promotes cell proliferation by regulating the Wnt signaling pathway. **A** CCK-8 assay was performed to assess cell viability in the ANXA9 siRNA group or SKL2001 + ANXA9 siRNA group. **B** Transwell assays were performed to assess cell invasion in the ANXA9 siRNA group or SKL2001 + ANXA9 siRNA group. **C** EdU assays were performed to assess cell proliferation in the ANXA9 siRNA group or SKL2001 + ANXA9 siRNA group. **D** Western blot was performed to examine protein levels in the ANXA9 siRNA group or SKL2001 + ANXA9 siRNA group. *P < 0.05, **P < 0.01

β-catenin is a key functional effector molecule of the Wnt signaling pathway, and is crucial in tumorigenesis and development. To confirm whether ANXA9 modulates Wnt/β-catenin, we evaluate the effect of ANXA9 on the nuclear translocation of β-catenin. The subcellular fractionation results showed that ANXA9 siRNA could decrease the nuclear β-catenin levels in HT29 and HCT116 cells (Fig. S3a). Immunofluorescence staining further showed that ANXA9 siRNA could decrease the nuclear β-catenin level (Fig. S3b). EMT is closely related to tumor growth and metastasis. We also used western blot to evaluate the expression levels of related proteins after ANXA9 knockdown. Knockdown of ANXA9 significantly reduced Snail and Vimentin levels, and enhanced E-cadherin levels in HT29 and HCT116 cells (Fig. 6E). Moreover, the results of immunohistochemical staining showed that ANXA9 expression level positively correlated with the expression level of vimentin (Fig. S3c). SKL2001 is an activator of the Wnt signaling pathway. We found that ANXA9 siRNA inhibited cell proliferation and invasion while SKL2001 treatment reversed the biological effects of ANXA9 knockdown (Fig. 7A–C). Moreover, SKL2001 restored Wnt pathway activity which was inhibited by ANXA9 knockdown (Fig. 7D). The data indicate that the growth-promoting effect of ANXA9 on CRC cells are mediated through the Wnt signaling pathway.

## Discussion

ANXA family members play essential roles in several types of cancers. For example, ANXA6 and ANXA7 are reported to act as tumor suppressor genes in gastric cancer [19] and prostate cancer [20]. In contrast, ANXA1 and ANX10 are reported to be significantly up-regulated and to act as oncogenes in papillary thyroid carcinoma [21] and extrahepatic cholangiocarcinoma [22]. Zhang et al*.* reported that ANXA9 expression is different in different cancer types [23]. In this study, we used TCGA and GEPIA databases to analyze the expression of ANXA family members in CRC samples. We confirmed that ANXA2/3/4/9 mRNA expression is higher in CRC tissue than in normal tissue. Based on survival curve analysis, only ANXA9 expression was associated with the prognosis of patients with CRC.

We also found that ANXA9 expression is significantly increased in CRC tissues and cells. ANXA9 expression was closely correlated with a number of clinical features in CRC, including age, pathological stage, TNM stage, and OS event status. Our findings are consistent with a previous report which evaluated 105 paired clinical samples and found that ANXA9 was elevated in CRC and correlated with clinicopathological parameters and poor prognosis [12]. Collectively, these results indicate that ANXA9 acts as an oncogene in the CRC.

The tumor microenvironment (TME) has significant impacts on cancer survival and development (TME). In addition to the tumor cells themselves, the TME also includes surrounding blood vessels, the extracellular matrix, fibroblasts, immune and inflammatory cells, bone marrow-derived suppressor cells, and signaling molecules, all of which can affect cancer occurrence and development [24]. To our knowledge, the correlation between ANXA9 and the levels of immune infiltration in the tumor microenvironment of colorectal cancer has not been reported. Thus, we investigated the correlation between the expression of ANXA9 and immune cell infiltration. We found that ANXA9 expression was positively related to the abundance of Tcm, and negatively associated with the abundance of Th2 cells, cytotoxic cells, aDC, T cells, Th1 cells, Treg, B cells, DC cells, NK CD56bright cells, TFH, T helper cells, CD8 T cells, neutrophils, iDC, macrophages, and mast cells. ANXA9 was negatively correlated with ImmuneScore and EstimateScore, not with StromalScore. ANXA9 expression level inversely related to the expression level of CD3 and CD68. Although the relationship between ANXA9 and immune infiltration has not been well studied, the relationship between other ANXA family members and immune infiltration has been extensively reported. For example, ANXA1 promotes the suppressive functions of Tregs in triple-negative breast cancer [7], ANXA2 acts as a potential marker of immunosuppression in glioma [25], and ANXA5 expression in stomach adenocarcinoma is significantly correlated with various infiltrating immune cells [26]. These suggest that ANXA9, as a member of ANXA family, may affect the immune response in CRC, but the effects of ANXA9 in regulating immune-infiltrating cells requires further investigation.

Bioinformatic analysis suggests that the ANXA9 may function as a potential therapeutic target in CRC. However, this merits validation through rigorous experimentation. Therefore, we investigated the effects of ANXA9 on the biological behavior of CRC. Consistent with a previous report [12], ANXA9 knockdown inhibited the proliferation, migration, invasion, and cell cycle progression of CRC cells. We also explored possible molecular mechanisms through which ANXA9 exerts its protumor effects in CRC. We found that the genes co-expressed with ANXA9 are enriched in the PI3K-AKT pathway, the Wnt signaling pathway, and amyotrophic lateral sclerosis. The Wnt/β-catenin pathway is involved in cell proliferation, cell survival and differentiation [27], and most colorectal cancers are caused by β-catenin gene mutations [28]. Therefore, we evaluated whether ANXA9 affects the Wnt pathway. The subcellular fractionation results confirmed that ANXA9 siRNA could decrease the nuclear β-catenin levels in HT29 and HCT116. Immunofluorescence staining further confirmed that ANXA9 siRNA could decrease the nuclear β-catenin level in HT29 and HCT116 cells. Through western blot, ANXA9 knockdown inhibited β-catenin protein expression. Furthermore, we found that the Wnt activator SKL2001 could reverse the effects of ANXA9 knockdown. However, the precise mechanism by which ANXA9 affects the Wnt pathway in CRC requires further investigation.

In current study, we demonstrated that ANXA9 promotes cell proliferation by regulating the Wnt signaling pathway and affect the immune response in CRC. However, the study has several limitations. First, although we confirmed the effect of ANXA9 in vitro, it needs in vivo experiments to further verify the function of ANXA9 in CRC. Second, the number of clinical samples is small and more samples are needed for verification. In addition, the effects of ANXA9 in regulating immune response requires further investigation.

In summary, we found that ANXA9 expression is significantly increased in CRC compared to normal tissue, and high ANXA9 expression is associated with poor prognosis in CRC. Furthermore, ANXA9 expression correlated with adverse clinical features in CRC. Finally, functional enrichment analysis and in vitro experiments showed that ANXA9 promotes CRC cell proliferation by regulating the Wnt signaling pathway. Collectively, these data reveal the potential role of ANXA9 in CRC, which will help us to better understand the role of ANXA9 in tumorigenesis and development of CRC, and suggests that ANXA9 could serve as a prognostic biomarker for CRC.

## Supplementary Information

Below is the link to the electronic supplementary material.

Supplementary file1 (DOCX 23 KB)

Supplementary file2 (DOCX 3257 KB)

## Data Availability

The data generated in the present study are included in the figures and/or tables of this article.
