# Supplementary material for: Annexin A9 promotes cell proliferation by regulating the Wnt signaling pathway in colorectal cancer
Source: Hum Cell. 2023 Jun 22;36(5):1729–40. doi: 10.1007/s13577-023-00939-x (PMC10390359; doi:10.1007/s13577-023-00939-x)
Supplement: Supplementary file 1 — Supplementary file1 (DOCX 23 KB) [file 13577_2023_939_MOESM1_ESM.docx]

Table S1.Clinical characteristics of CRC patients in the ANXA9-high and the ANXA9-low expression groups.

| Characteristic | Low expression of ANXA9 | High expression of ANXA9 | p | Method | |  |
| --- | --- | --- | --- | --- | --- | --- |
| n | 309 | 310 |  |  |  |  |
| T stage, n (%) |  |  | 0.040 | Chisq.test | | |
| T1 | 15 (2.4%) | 5 (0.8%) |  |  | | |
| T2 | 56 (9.1%) | 49 (7.9%) |  |  | | |
| T3 | 209 (33.9%) | 213 (34.5%) |  |  | | |
| T4 | 28 (4.5%) | 42 (6.8%) |  |  | | |
| N stage, n (%) |  |  | 0.127 | Chisq.test | | |
| N0 | 187 (30.4%) | 164 (26.6%) |  |  | | |
| N1 | 73 (11.9%) | 77 (12.5%) |  |  | | |
| N2 | 49 (8%) | 66 (10.7%) |  |  | | |
| M stage, n (%) |  |  | 0.007 | Chisq.test | | |
| M0 | 239 (43.8%) | 220 (40.3%) |  |  | | |
| M1 | 31 (5.7%) | 56 (10.3%) |  |  | | |
| Gender, n (%) |  |  | 0.244 | Chisq.test | | |
| Female | 152 (24.6%) | 137 (22.1%) |  |  | | |
| Male | 157 (25.4%) | 173 (27.9%) |  |  | | |
| Age, n (%) |  |  | 0.038 | Chisq.test | | |
| <=65 | 121 (19.5%) | 148 (23.9%) |  |  | | |
| >65 | 188 (30.4%) | 162 (26.2%) |  |  | | |
| Pathologic stage, n (%) |  |  | 0.015 | Chisq.test | | |
| Stage I | 59 (9.8%) | 46 (7.7%) |  |  | | |
| Stage II | 122 (20.4%) | 105 (17.5%) |  |  | | |
| Stage III | 90 (15%) | 89 (14.9%) |  |  | | |
| Stage IV | 31 (5.2%) | 57 (9.5%) |  |  | | |
| Primary therapy outcome, n (%) |  |  | 0.780 | Fisher.test | | |
| PD | 15 (5.1%) | 18 (6.1%) |  |  | | |
| SD | 2 (0.7%) | 3 (1%) |  |  | | |
| PR | 8 (2.7%) | 7 (2.4%) |  |  | | |
| CR | 131 (44.1%) | 113 (38%) |  |  | | |
| Lymphatic invasion, n (%) |  |  | 0.838 | Chisq.test | | |
| No | 165 (29.6%) | 166 (29.7%) |  |  | | |
| Yes | 116 (20.8%) | 111 (19.9%) |  |  | | |
| OS event, n (%) |  |  | 0.049 | Chisq.test | | |
| Alive | 256 (41.4%) | 236 (38.1%) |  |  | | |
| Dead | 53 (8.6%) | 74 (12%) |  |  | | |
| DSS event, n (%) |  |  | 0.091 | Chisq.test | | |
| Alive | 270 (45.2%) | 249 (41.7%) |  |  | | |
| Dead | 32 (5.4%) | 46 (7.7%) |  |  | | |
| Age, median (IQR) | 68 (59, 78) | 66 (57, 74) | 0.017 | Wilcoxon | | |
