# Supplementary material for: Annexin A9 promotes cell proliferation by regulating the Wnt signaling pathway in colorectal cancer
Source: Hum Cell. 2023 Jun 22;36(5):1729–40. doi: 10.1007/s13577-023-00939-x (PMC10390359; doi:10.1007/s13577-023-00939-x)
Supplement: Supplementary file 2 — Supplementary file2 (DOCX 3257 KB) [file 13577_2023_939_MOESM2_ESM.docx]

**
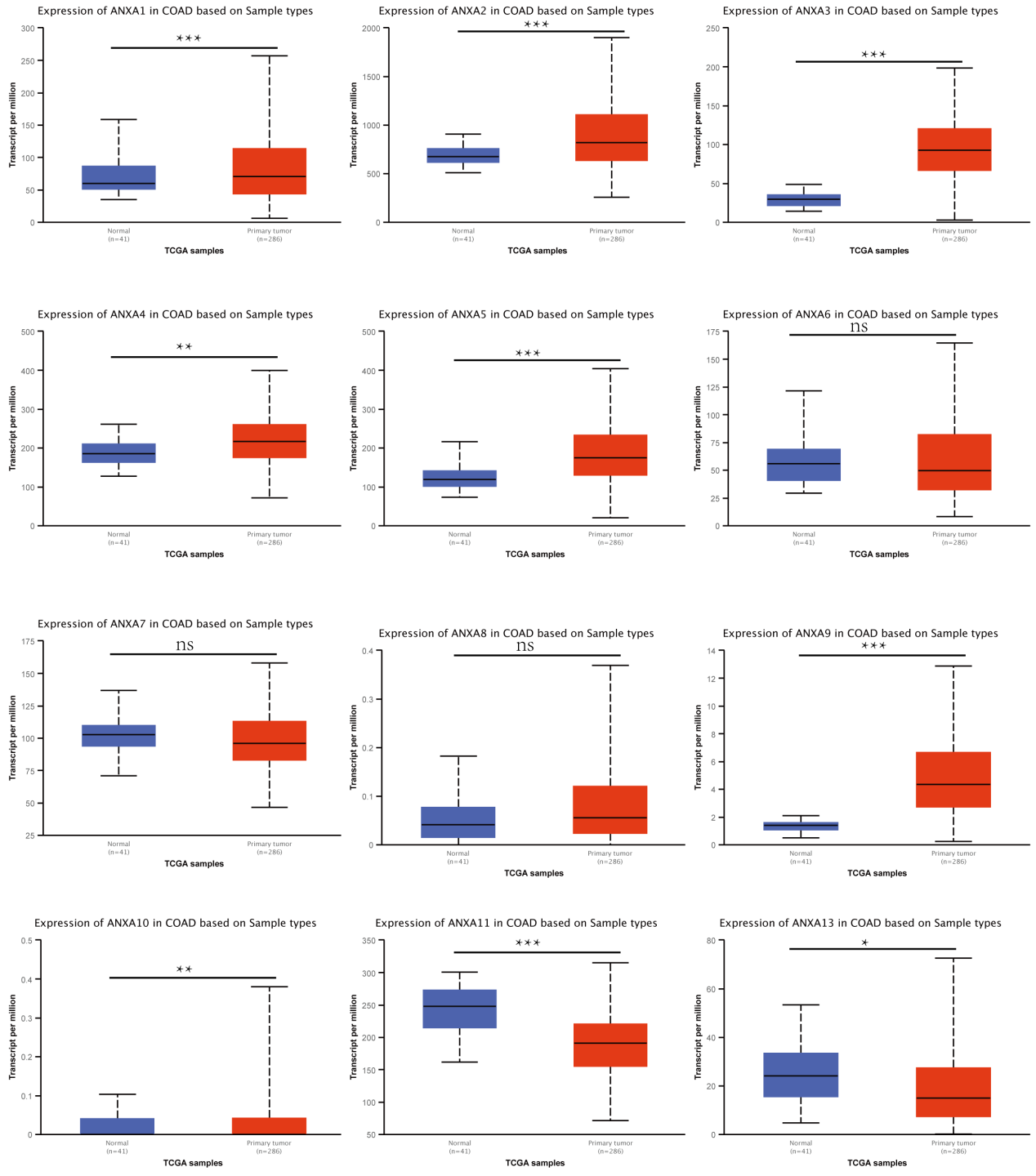
**

**Figure S1. mRNA expression levels of ANXA family genes in CRC by UCLCAN.** * P < 0.05, ** P < 0.01, ***P < 0.001.


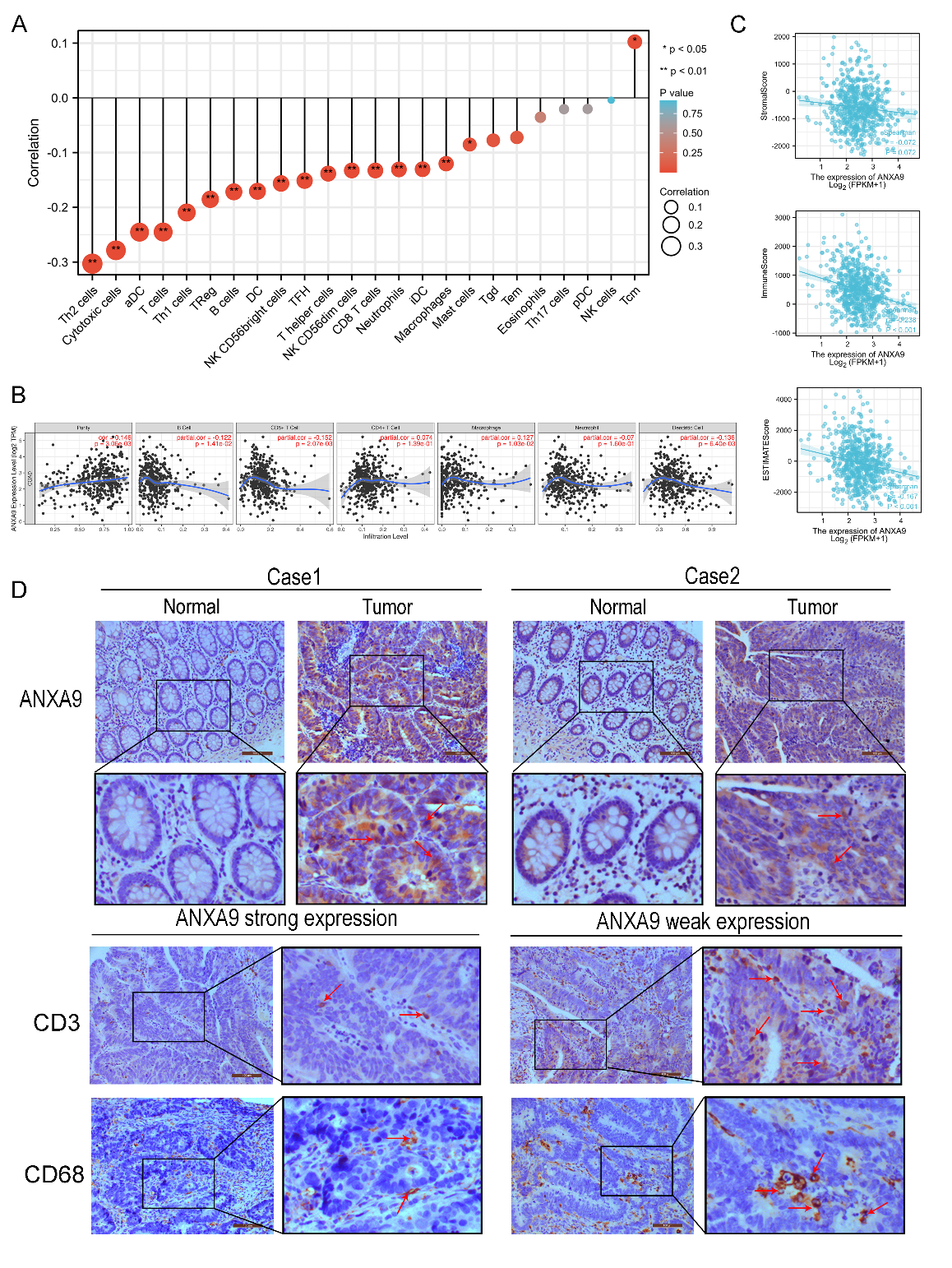


**Figure S2. Correlation between ANXA9 expression and immune infiltration levels in CRC.** (A) The lollipop diagram shows the relationship between the relative abundance of 24 immune cells and the expression of ANXA9. * P < 0.05, ** P < 0.01. (B) The correlation between ANXA9 expression and immune infiltration in the TIMER database. (C) The correlation between ANXA9 expression and immunescore, estimatescore and stromalscore by scatter plots. (D) Immunohistochemical analyses analysis of ANXA9, CD3 and CD68 levels in CRC tissues.


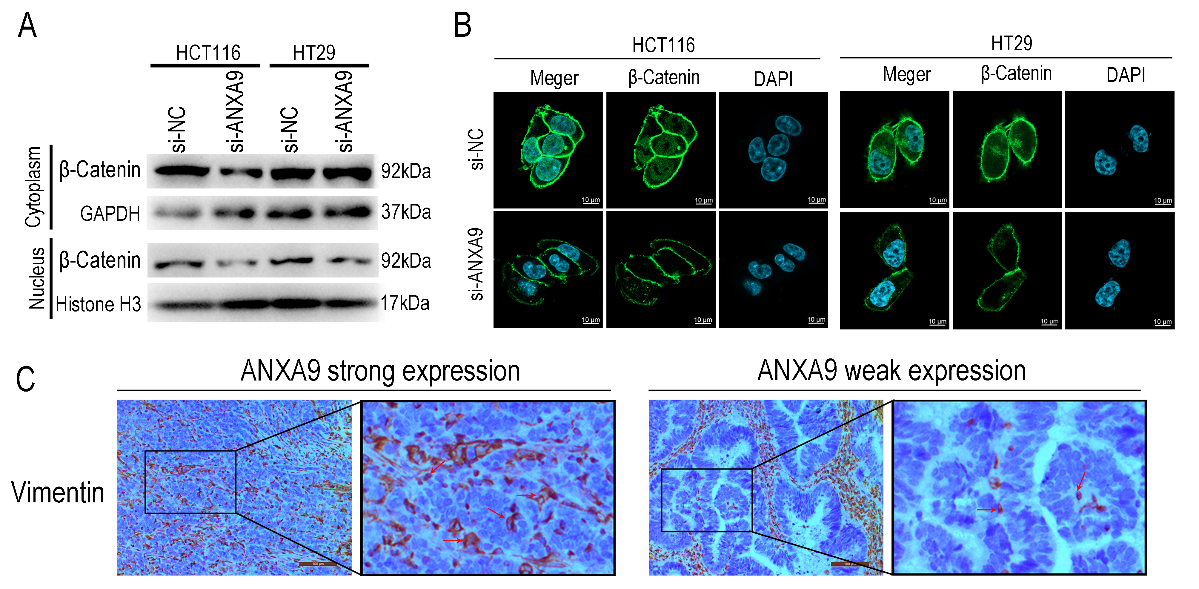


Figure S3. (A) Nuclear fractions were analyzed by WB analysis after ANXA9 knockdown. (B) Subcellular β-catenin localization in indicated cells was assessed by immunofluorescence staining. (C) Immunohistochemical analyses analysis of Vimentin protein levels in CRC tissues with ANXA9 strong expression or ANXA9 weak expression.
